# Folate deficiency and utilization of folic acid fortified flour among pregnant women attending antenatal clinic at Pumwani Maternity Hospital, Kenya, 2015

**DOI:** 10.11604/pamj.supp.2017.28.1.9296

**Published:** 2017-11-04

**Authors:** Elizabeth Mgamb, Zeinab Gura, Peter Wanzala, Jane Githuku, Anselimo Makokha

**Affiliations:** 1 Jomo Kenyatta University of Agriculture and Technology, Nairobi, Kenya; 2 Field Epidemiology and Laboratory Training Program-Kenya, Kenya; 3 Kenya Medical Research Institute, Kenya

**Keywords:** Folate deficiency, folic acid, utilization

## Abstract

**Introduction:**

in 2012, the Government of Kenya amended the Food, Drug and Chemical Substances Act to make the fortification of maize and wheat flour with folic acid mandatory. We assessed folate deficiency, awareness and use of folic acid fortified flour among pregnant women receiving antenatal care (ANC) at a clinic at Pumwani Maternity Hospital, Kenya, 2015.

**Methods:**

we conducted a cross-sectional survey at Pumwani Maternity Hospital between October and November 2014. We enrolled pregnant women who received ANC and interviewed them using a semi-structured questionnaire after obtaining informed consent. Blood samples were collected from all study participants and serum folate level was analyzed by electrochemiluminescence immunoassay. Folate deficiency was defined as serum folate of < 10nmols/L and borderline folate deficiency was defined as serum folate of between 10nmols/L and 15nmols/L.

**Results:**

among the 247 study participants, two (1%) had folate deficiency. One hundred and seventy-nine (73.4%) had heard about folic acid, but only 56 (23%) had heard about folic acid fortified flour. Overall, 198 (80%) study participants consumed fortified brands of maize flour and 205 (84%) consumed fortified brands of wheat flour; only four (2%) and two (1%) of study participants consumed specific brands of maize and wheat flour respectively because they were fortified.

**Conclusion:**

the prevalence of folate deficiency was low and this may have been because of the availability of fortification programs. Although there was limited knowledge of fortified flour, utilization was high. The Kenyan Ministry of Health should enforce implementation of the legislation on maize flour and wheat flour fortification by all milling industries.

## Introduction

Folate deficiency is one of the micronutrient deficiencies of global public health concern, especially among women of child bearing age [1]. Folate deficiency in pregnant women increases the risk of neural tube defects (NTDs), premature births, intrauterine growth retardation, congenital heart defects and oro-facial cleft defects in newborns [2,3]. The protective effects of adequate maternal folate consumption on the risk of having a pregnancy affected with NTDs have been demonstrated in observational and interventional studies. A systematic review of 27 studies which assessed the changes in prevalence of NTDs as a result of mandatory folic acid food fortification reported a decline in the NTD prevalence following mandatory fortification with the greatest declines seen in Costa Rica (58% decline), Argentina (50% decline) and Canada (49% decline) [3].

The global prevalence of folate deficiency in the population is unknown because of a lack of data from many parts of the world [1]. Many countries, especially in low income countries, do not routinely assess folate status of the population [4]. In countries that do have data on folate status, reported folate deficiency data vary because of the use of different testing methods, varying cut-off ranges and heterogeneity by race or ethnic group or by geographical region [5]. One Canadian study during the post-mandatory fortification period among pregnant women yielded a prevalence of 1% folate deficiency [6]. In South Africa, a study comparing the folate status among women of reproductive age in the pre-fortification versus post-fortification periods showed a reduction in the prevalence of folate deficiency from 27.6% to 0% [7]. High prevalence of folate deficiency among women of reproductive age were reported in studies conducted before implementation of mandatory fortification of flour in Ethiopia (46%) and Benin (31%) [8,9]. However, there is limited information on the prevalence of folate deficiency in Kenya.

Global data on awareness and utilization of folic acid is limited and is mostly from small studies conducted in various countries. Some of these studies have reported high awareness about folic acid while others have reported low awareness. High levels of awareness on folic acid among women of reproductive age was found in studies in the U.S. states of Kansas (89%) and Texas (78%) [10,11]. Folic acid awareness levels among pregnant women receiving antenatal care was also found to be high (88%) in Australia [12]. In Nigeria, a high level of awareness (64.6%) about folic acid among pregnant women seeking antenatal care in one of the major hospitals was reported [13]. Studies in Chile reported low folic acid awareness of 47% [14]. The utilization of folic acid supplements by pregnant women was found to be 25% and 15%, respectively, in studies conducted in USA and Chile [10,14].

In Kenya, two interventions, folate and iron supplements for individual consumption and legislation for mandatory fortification of milled maize and wheat flour have been used to improve the folate status in the population. Iron-folate supplements are usually given to women during the first antenatal care (ANC) visit (below 16 weeks gestation) with the goal of preventing anemia, but these do not reduce risk of NTDs, because the neural tubes closes between 21 and 28 days after conception, before most women know that they are pregnant [15]. The Kenya Food, Drug and Chemical Substances Act was amended in 2012 to include mandatory folic acid fortification of maize and wheat flour [16]. Media campaigns promoting the use of fortified flour have been conducted to raise awareness and promote demand for fortified flour since 2012. However, there is limited information on the level of awareness and utilization of fortified flour in the country, nor is there an estimate of the prevalence of folate deficiency. The study determined the prevalence of folate deficiency and the levels of awareness and use of folic acid among pregnant women seeking prenatal services at a high-volume birthing hospital in Nairobi, Kenya.

## Methods

### Study design and site

A cross-sectional study was conducted between October and November 2014 at Pumwani Maternity Hospital. Located in Nairobi County, Kenya, Pumwani Maternity Hospital is both a referral facility for complicated obstetric cases and a primary birthing hospital for women who live proximal to it with approximately 17,000 deliveries annually.

### Inclusion and exclusion criteria

All pregnant women 18 years of age and older presenting at Pumwani maternity for their first ANC visit who gave informed consent were included in the study. Those pregnant women with pregnancies of gestational age >28 weeks were excluded from the study.

### Sample size and sampling

The sample size was calculated using the Cochran formula [17] with the assumptions of 31% prevalence of folate deficiency based on a study in Benin [9] and assuming a 95% confidence level. This yielded a sample size of 259 after accounting for an expected 10% non-response rate. Pregnant women attending ANC were systematically sampled. To calculate the sampling interval, we first obtained the number of pregnant women aged 18 years and older coming to Pumwani maternity hospital for their first ANC visit through a review of ANC records for the two months preceding the study period (633 women). This was then divided by the sample size, which gave a sampling interval of three. Using the daily attendance register as the sampling frame, the first study participant was randomly selected and subsequent participants were selected according to the sampling interval. If an eligible person declined to participate in the study, the next listed client in the daily attendance register was selected as their replacement.

### Collection of data and blood samples

A pre-tested, structured questionnaire was used to interview the study participants. We collected data on: socio-demographic, obstetric and clinical characteristics as well as information on awareness and use of folic acid supplements and folic acid fortified flour. Information on diet was also collected using food frequency questions and 24-hour recall of food items consumed. Collection of blood samples was done by trained laboratory technologists. Two milliliters of blood were collected from each participant using a sterile anticoagulant free tube. The tubes were labeled with questionnaire number, date and time of sample collection. All the samples collected during the day were placed in a disposable plastic bag lined with absorbent material, placed in a cool box at a temperature of 2-8°C and transported to the laboratory. Analyses of blood samples were done daily by electrochemi-luminescence immunoassay [18]. Folate deficiency was defined as serum folate levels < 10 nmol/L (4 ng/mL), borderline folate deficiency was defined as serum folate levels between 10nmol/L (4ng/mL) and 15nmol/L (6ng/mL) and normal folate was defined as serum folate levels > 15nmol/L (6ng/mL) [5].

### Data management and analysis

Data were entered and cleaned using EPI INFO version 7© and analysis was conducted using SPSS © version 16.0. Descriptive statistics were calculated as frequencies and proportions for categorical variables and mean or median for continuous variables. We compared means using the t test, p value of < 0.05 was considered to be significant.

### Ethical considerations

A written informed consent was obtained from all the study subjects after fully explaining to them the nature of the study. Ethical approval was sought from the Kenyatta National Hospital Research and Ethics Board, Number P481/8/2014.

## Results

### Socio-demographic characteristics

We enrolled 259 women, with ages ranging from 15 to 43 years. Twelve blood samples were of insufficient quantity and were not included, therefore serum folate results were available for 247 participants. The socio-demographic characteristics of the study participants are summarized in Table 1.

**Table 1:** socio-demographic characteristics of pregnant women seeking care at Pumwani Maternity Hospital, October 2015 to November 2015

Characteristic	Categories	Number (%)
Age	<20	11 (4.5)
	20-29	148 (59.9)
	30-49	88 (35.6)
Religion	Christian	220 (89.1)
	Muslim	27 (10.9)
Residence	Informal settlement	73 (29.6)
	Formal settlement	174 (70.4)
Level of education	Tertiary	65 (26.3)
	Secondary	113 (45.7)
	Primary	67 (27.1)
	None	2 (0.8)
Employment Status	Formal employment	63 (25.5)
	Self employed	109 (44.1)
	Unemployed	75 (30.4)
Marital status	Married	196 (79.4)
	Single	49 (19.8)
	Divorced	2 (0.8)
Spouse’s level of Education	Tertiary	68 (27.5)
	Secondary	96 (38.9)
	Primary	29 (11.7)
	None	1 (0.4)
	Don’t Know	53 (21.5)
Spouse’s Employment Status	Formal employment	104 (42.1)
	Self employed	93 (37.7)
	Unemployed	2 (0.8)
	Don’t Know	48 (19.4)
**Total (n)**		**247**

### Prevalence and characteristics of folate deficiency participants

**Figure 1:**
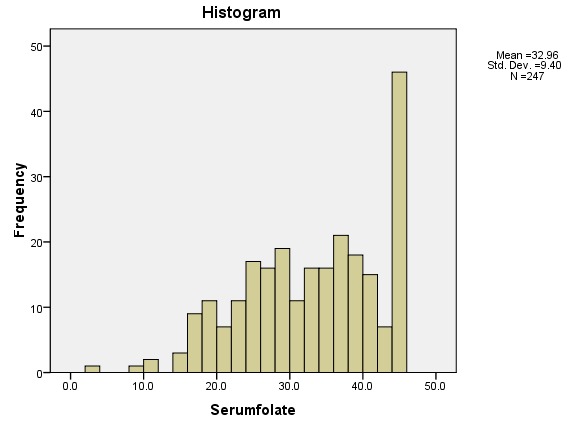
folate level distribution of study participants, Pumwani Maternity Hospital, October 2015 to November 2015

The median folate levels were 33.7 nmols/l (IQR 14.5). The folate distribution is shown in Figure 1. Out of the 247 women, two (0.8%) had folate deficiency, three (1.2%) had borderline folate deficiency and 242 (97%) had normal folate levels. The two participants with folate deficiency were aged 22 and 30 years, had secondary education, were from the same area of residence which was non-slum, had no chronic illnesses, were not on any medication, reported not taking alcohol or smoking cigarettes, were not aware of folic acid fortified flour but consumed folic acid fortified flour and one reported using oral contraceptives. Of the three who had borderline folate deficiency; their ages were between 19 and 27 years, two had secondary education, all were from non slum residence, their gestation was between 15 and 23 weeks, all reported oral contraceptives use, two had chronic illnesses (HIV and epilepsy) and were on medication, one reported smoking cigarettes, all the three reported consuming alcohol, all had never heard of folic acid fortified flour and two consumed fortified flour

### Awareness on folic acid

Out of the 247 women, 179 (73%) had heard about folic acid but only half (90 women, 50%) could mention one or more of the benefits of folic acid to women of reproductive age and pregnant women. Among those who had heard about folic acid, the sources of information were: television 77% (137/179), radio 25% (44/179), health care worker 11% (20/179) and neighbour 0.6% (1/179). Only 56 (23%) of the participants were aware of folic acid fortified flour. The sources of information on fortified flour were: television 43% (24/56), friends 27% (15/56), radio 13% (7/56) and health care worker 7% (4/56), as indicated in Table 2.

**Table 2:** obstetric characteristics of pregnant women seeking care at Pumwani Maternity Hospital, October 2015 to November 2015

Variable	Categories	Frequency (%) N=247
Gestation in weeks	≤13 weeks	84(34.0)
	>13 weeks	163(66.0)
Gravidity	1	84(34.0)
	2	72(29.1)
	3	63(25.5)
	4	20(8.1)
	5+	8(3.2)
Ever used any family planning method	No	47(19.0)
	Yes	200(81.0)
Pregnancy planned	No	101(40.9)
	Yes	146(59.1)

### Utilization of folic acid supplements, fortified flour and dietary intake

Only 33 (13%) of the study participants reported that they were using folic acid supplements. The mean serum folate levels among those who reported using folic acid was 35.4mmols/l while that of those who reported not using folic acid supplements was 32.7mmols/l (t240 = 1.563, p = 0.119). Most women (88%) reported that their main source of maize flour was purchased as flour produced from large-scale commercial milling industries, while the remainder (12%) obtained their maize flour from small-scale local mills. All participants reported that their main source of wheat flour was purchased as flour produced from milling industries. One hundred and ninety-eight participants (80%) reported using folic acid fortified maize flour and 205 (84%) participants reported using folic acid fortified wheat flour. Only four (2%) participants reported that they purchased particular brands of maize flour because they are fortified. Two hundred and five study participants (83%) reported purchasing specific brands of wheat flour because of their taste rather than fortification status 2(1%) as indicated in Table 3. The folate rich foods that were reported to have been consumed daily by the study participants included: bananas (56%), ugali (48%) and green vegetables (44%) (Table 4). The majority of participants reported having consumed at least one serving of fruits (73%), ugali (68%), wheat containing foods (63%) and vegetables (61%) the previous day.

**Table 3:** respondents’ awareness, knowledge and utilization of folic acid among pregnant women seeking care at Pumwani Maternity Hospital, October 2015 to November 2015

Section	Variables	Number n=259	Percentage
Awareness	Heard of folic acid supplements	189	73
	S**ource of information (n=189)**		
	Television	144	76.2
	Radio	47	24.9
	Health care worker	21	11.1
	Neighbor	1	0.5
	Heard of fortified flour	63	22.7
	**Source of Information (n=63)**		
	Television	28	44.4
	Radio	17	27.0
	Health care worker	8	12.7
	Neighbor/friends	4	6.3
Knowledge	Could mention at least one benefit of folic acid to pregnant women	90	34.7
	**Benefits mentioned (n=90)**		
	Prevents anemia	64	71.1
	Prevents delivery of Low birth weight babies	39	43.3
	Prevents birth defects	27	30.0
	Prevents prematurity	13	14.4
	Knew the meaning of fortified flour	42	17.0
	Knew how to identify fortified flour	38	15.4
Utilization of folic acid (supplements and fortified flour	Reported folic acid supplements use	38	14.7
	**Period started**		
	Peri-conceptionally	5	13.2
	First trimester	25	65.8
	Second trimester	7	18.4
	Consume maize flour from large scale millers	229	88.4
	Consume fortified maize flour brands	208	80.3
	**Reasons for buying particular maize flour brands**		
	Taste	147	59.5
	Availability	45	18.2
	Cost	2	0.8
	Fortified	4	1.6
	Others	24	9.7
	**Consume wheat flour from large scale millers**		
	Consume fortified wheat flour brands	259	100
	Reasons for buying particular wheat flour brands	216	83.4
	Taste	180	72.9
	Availability	26	10.5
	Cost	0	0.0
	Fortified	2	0.8
	Others	9	3.6

**Table 4:** frequency of consumption of folate rich foods among pregnant women seeking care at Pumwani Maternity hospital, October 2015 to November 2015

Food	Daily Frequency (%)	4-6 days a week Frequency (%)	1-3 days a week Frequency (%)	Once in 2 weeks Frequency (%)	Rarely Frequency (%)	Never Frequency (%)
Bananas	139 (54)	8 (3)	54 (21)	1 (0.4)	16 (6)	15 (6)
Ugali	119 (46)	41 (16)	75 (29)	0 (0)	3 (1)	0 (0)
Green vegetables	109 (42)	27 (10)	90 (35)	0 (0)	13 (5)	1 (0.4)
Oranges	72 (28)	11 (4)	54 (21)	2 (1)	74 (29)	27 (10)
Avocadoes	56 (22)	7 (3)	68 (26)	4 (2)	70 (27)	0 (0)
Pawpaw	30 (12)	9 (3)	39 (15)	4 (2)	92 (36)	63 (24)
Beans	20 (8)	9 (3)	134 (52)	9 (3)	50 (19)	18 (7)
Chapatis	17 (7)	11 (4)	102 (39)	42 (16)	60 (23)	0 (0)
Liver	4 (2)	2 (1)	61 (24)	14 (5)	96 (37)	58 (22)
Alcoholic beverages	0 (0)	0 (0)	1 (0.)	0 (0)	6 (2)	231 (89)

## Discussion

Our study was conducted two years after the Government of Kenya amended the Food, Drug and Chemical Substances Act to have mandatory fortification of maize flour and wheat flour with folic acid and other nutrients.

We identified very few pregnant women with folate deficiency (2%) attending Pumwani Maternity hospital. Although we found that awareness on folic acid was high among these women, knowledge about the fortification of flour with folic acid was low. Reported consumption of folic acid supplements (both peri-conceptionally and during pregnancy) was also low, although the reported use of folic acid fortified flour was high.

We believe that the low prevalence of folate deficiency in this study could be attributed to the implementation of mandatory fortification of maize and wheat flour. Our study found that all the participants were exposed to either fortified wheat flour or maize flour, and this could explain the low prevalence. In addition, most study participants reported consuming folate rich foods.

The low prevalence of folate deficiency in our study is consistent for a population of pregnant women seeking ANC following implementation of mandatory folic acid fortification. In South Africa, the prevalence of folate deficiency among non-pregnant rural women of child bearing age was found to have reduced from 27.9% to 0% nine months after mandatory fortification of maize and wheat flour was introduced [7]. In Canada, a national survey conducted following the start of mandatory fortification reported a prevalence of 1% among the Canadian women of reproductive age [6]. However, our findings differ from studies in three African countries conducted before mandatory fortification including a community based study in Ethiopia among women of child bearing age which found 46% of women deficient [8], a study in Benin among HIV negative pregnant women at the time of first ANC visit which found 31% of women folate deficient [9] and a study conducted in Eastern Sudan among pregnant women that found 57.7% of women folate deficient [19].

We found a high level of awareness on folic acid among women in our study, which could be caused by ongoing media advertisements about iron folic supplements during the study period, with television reported as the main source of information on folic acid. The proportion of women who had heard of folic acid from health care workers was low, therefore additional targeting of health care workers to discuss the importance of folic acid supplementation may be an important future strategy.

There was low uptake of folic acid supplements. This differs from a study conducted in the year 2013 in a referral hospital in central Kenya where the utilization of folic acid supplements was found to be 51.2% [20]. The difference in the uptake of folic acid supplements could be attributed to differences in target population. Our target population were women who were coming for the first ANC visit to a large referral hospital, whereas the central Kenya study included both initial ANC visits and subsequent revisits. In Kenya, most pregnant women start taking folic acid supplements after they are told to do so by health care providers during their initial ANC visit, hence utilization of folic acid supplements during subsequent visits is usually higher. There were low levels of awareness about folic acid fortified flour and few study participants who bought specific brands of maize and wheat flour specifically because of fortification, yet utilization of folic acid fortified flour was high. This is because most of the study participants consumed maize and wheat flour from large scale millers, most of which produced fortified flour following the legislation on mandatory fortification of wheat flour. This highlights the importance of mandatory fortification programmes in improving the folate status of the population.

Our study had several limitations: first, this was only a two months study and there could be differences in prevalence of folate deficiency and utilization of folate fortified flour over a longer period. Second, it was conducted in one referral hospital in a capital of Nairobi, therefore not generalizable to all of Kenya. Third, we did not collect data on consumption of other fortified flour products like bread on the 24 hours recall on consumption of folate rich foods and data on quantities consumed was also not collected. Fourth, this was self-reported data and this may lead to recall bias. At the time of the survey, there was no surveillance for neural tube defects but since then, Birth Defect Surveillance has started in the same hospital, which could allow for future comparisons on the effect of folic acid fortification.

## Conclusion

The prevalence of folate deficiency among pregnant women attending Pumwani Maternity Hospital was low possibly because of the implementation of mandatory fortification of maize and wheat flour. There were low levels on the knowledge of fortified flour although the utilization was high. There is need to reinforce the implementation of the legislation on maize flour and wheat flour fortification.

## What is known about this topic

- Folate deficiency increases the risk of neural tube defects (NTDs), prematurity, intra uterine growth retardation, congenital heart defects and oro-facial cleft defects in newborns;
- The prevalence of folate deficiency varies across different countries and decreases following mandatory fortification of specific staple foods with folic acid.

## What this study adds

- The study provides an estimate on the prevalence of folate deficiency in Kenya in one large referral hospital;
- It provides information on the levels of awareness and utilization of folic acid supplements and folic acid fortified flour.

## Competing interests

The authors declare no competing interest.
